# Development and validation of tumor‐size‐stratified prognostic nomograms for patients with uterine sarcoma: A SEER database analysis

**DOI:** 10.1002/cam4.5014

**Published:** 2022-07-16

**Authors:** Shiyu Cao, Xianzhen Liao, Kekui Xu, Haifan Xiao, Zhaohui Shi, Yanhua Zou, Can Li, Yingyun Hu, Shipeng Yan

**Affiliations:** ^1^ Department of Cancer Prevention and Control Hunan Cancer Hospital and the Affiliated Cancer Hospital of Xiangya School of Medicine, Central South University Changsha Hunan Province China

**Keywords:** nomogram, prognosis, SEER, survival

## Abstract

**Background:**

Tumor‐size‐stratified analysis on the prognosis of uterine sarcoma is insufficient. This study aimed to establish the tumor‐size‐stratified nomograms to predict the 3‐ and 5‐year overall survival (OS) of patients with uterine sarcoma.

**Methods:**

The data analyzed in this study were obtained from the Surveillance, Epidemiology, and End Results (SEER) database. We collected data from patients with uterine sarcoma diagnosed between 2004 and 2015. According to the median tumor size of 7.8 cm, the enrolled patients were divided into two tumor size (TS) groups: TS <7.8 cm and TS ≥7.8 cm. Patients in each group were randomly divided into the training and validation cohorts with a ratio of 7:3. Chi‐square test was used to compare differences between categorical variables. Multivariate Cox regression models were used to identify significant predictors. We calculated the concordance index (C‐index) and the area under the receiver operating characteristics curve (AUC) to validate the nomograms.

**Results:**

Compared with TS <7.8 cm group, TS ≥7.8 cm group had more patients of 45–64 years group, higher black race prevalence, higher proportion of myometrium tumor, higher stage, and higher grade; In the TS <7.8 cm training cohort, six variables (age, race, marital status, tumor primary site, stage, and grade) were identified as significantly associated with OS in multivariate analysis. However in the TS ≥7.8 cm training cohort, only four variables (surgery on primary site, tumor size, stage, and grade) were significantly identified; The C‐index of two nomograms were 0.80 and 0.73 in training cohorts, respectively, and the AUC values for 3‐ and 5‐year OS predictions in training cohorts were all above 0.80. Similar results were observed in validation cohorts.

**Conclusions:**

This study found that the significant prognostic factors were different between two tumor size groups of uterine sarcoma patients. The tumor‐size‐stratified nomograms, which we constructed and validated, might be useful to predict the probability of survival for patients with uterine sarcoma.

## Graphical abstract

This study aimed to establish the tumor‐size‐stratified nomograms based on the Cox proportional hazard regression model to predict the 3‐ and 5‐year overall survival of patients with uterine sarcoma. The nomograms established for patients in two tumor size groups showed satisfactory discriminative ability.

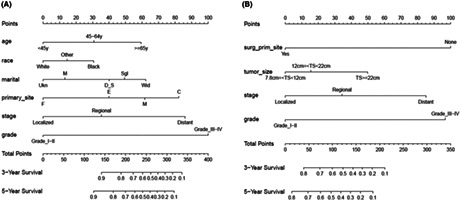

## 1 INTRODUCTION

Uterine sarcoma is a rare and diverse group of neoplasms, which is further classified into leiomyosarcoma, endometrial stromal sarcoma, adenosarcoma, and undifferentiated sarcoma.^1^ Research showed uterine cancer ranks sixth for incidence in women worldwide in 2018.^2^ The incidence of uterine sarcoma is 3%–7% of all uterine cancers.^3^ Compared with endometrial carcinoma, uterine sarcoma is highly aggressive and has a poorer prognosis. Early detection and diagnosis of uterine sarcoma are essential to improve the prognosis of patients. To date, the etiology of uterine sarcoma has not been understood completely. Several underlying factors, such as estrogen supplement, tamoxifen use, obesity, and diabetes are possibly associated with increased risks of uterine sarcoma.^3^, ^4^

Nomogram is a pictorial predictive model that has the ability to integrate the contribution of each prognostic variable on the result of prediction.^5^ Because of its numerous advantages compared to the Tumor‐Node‐Metastasis (TNM) staging system and the International Federation of Gynecology and Obstetrics (FIGO) staging system, the nomograms are widely used to evaluate the prognosis of patients with tumor in recent years.^5^, ^6^, ^7^

In recent years, different researches attempted to identify the prognostic factors in patients with uterine sarcoma. Tumor size is an important clinical factor with considerable prognostic value for many tumors, including uterine sarcoma.^8^, ^9^, ^10^, ^11^ Meanwhile, the appropriate prognostic predictors of predictive model may differ between patients with large tumor size and those with comparatively small tumor size. In order to predict the overall survival of patients with uterine sarcoma, the previous study usually established one nomogram for all tumor size groups. To date, tumor‐size‐stratified analysis on the prognosis of uterine sarcoma is insufficient. Using population‐based data from the Surveillance, Epidemiology, and End Results (SEER) database, a U.S. cancer database that covers approximately 48% of the U.S. population,^12^ this study aimed to establish the tumor‐size‐stratified nomograms based on the Cox proportional hazard regression model to predict the 3‐ and 5‐year overall survival of patients with uterine sarcoma.

## 2 MATERIALS AND METHODS

This study is about a data‐mining process using medical public database. The data‐mining process is usually divided into the following steps^13^: (1) select an appropriate database; (2) extract, clean, and convert data; (3) extract potentially useful information hidden in data using statistical methods or models; and (4) evaluate the models and results. The detailed process is as follows.

### 2.1 Data collection

Database technology is a software science that studies, administers, and utilizes databases. The data stored in the database are mined and analyzed by learning the basic theory and application methods of the database.^14^ All the data analyzed in this study were obtained from the SEER database, a data library supported by the US National Cancer Institute. In our study, all the data extraction processes were implemented by the SEER*Stat software version 8.3.9.2 (seer.cancer.gov/seerstat) after signing the user agreement, with the account number of 17573‐Nov2020.

We collected data from female patients with uterine sarcoma diagnosed between 2004 and 2015. The inclusion criteria included (1) primary site record: C54.1‐C54.3, C54.9, according to the Third Edition of International Classification of Diseases for Oncology (ICD‐O‐3); (2) histology type: 8800/3–8950/3, according to ICD‐O‐3; (3) complete survival months; (4) diagnostic confirmation was the positive histology and was no autopsy or a death certificate; (5) uterine sarcoma was the only primary tumor. The exclusion criteria included (1) patients with unknown information, such as race, stage, grade, tumor size (2) survival time <1 month. Eventually, a total of 1017 patients were included after the screening.

### 2.2 Variables and groups

The variables included for statistical analysis were as follows: demographics (age and race), marital status, tumor primary site, surgery on primary site, tumor size, tumor stage, grade, survival time, and vital status. In our study, age at diagnosis was grouped into <45, 45–64, and ≥ 65 years old. Race was classified into White, Black, and Other (including American Indian/AK Native, Asian/Pacific Islander). Marital status was categorized into married, single, divorced and separated, widowed, and unknown. Primary site was classified into four subgroups: endometrium, myometrium, fundus uteri, and corpus uteri. Patients were divided into two groups (none/yes) according to whether surgery on primary site had been done. Tumor size (TS) was classified as TS <7.8 cm and TS ≥7.8 cm. Then, TS <7.8 cm was classified into three subgroups: TS <2 cm, 2 cm ≤ TS <5 cm, and 5 cm ≤ TS <7.8 cm; TS ≥7.8 cm was also classified into three subgroups: 7.8 cm ≤ TS <12 cm, 12 cm ≤ TS <22 cm, and TS ≥22 cm. Stage fell into three categories: localized, regional, and distant. The degree of histologic differentiation of the uterine sarcoma was classified into four grades: grade I, well differentiated; grade II, moderately differentiated; grade III, poorly differentiated; and grade IV, undifferentiated or anaplastic.

According to the median tumor size of 7.8 cm, the enrolled patients were divided into two groups: TS <7.8 cm (*n* = 508) and TS ≥7.8 cm (*n* = 509). To establish and validate two separate nomograms, patients in each group were randomly divided into the training and validation cohorts with a ratio of 7:3.

### 2.3 Statistical analysis

In our study, the chi‐square test was used to compare differences between categorical variables. Overall survival (OS) was assessed to evaluate the prognosis and outcomes and was defined as the time from diagnosis of uterine sarcoma to death of any cause. Patients that survived until the end of follow‐up were treated as censors. Multivariate Cox proportional hazards regression models were used to identify significant predictors for OS of uterine sarcoma, and the results were presented as hazard ratios (HR) with corresponding 95% confidence intervals (CIs).

Significant variables (*P* < 0.05) found in the multivariate Cox regression analysis were input into the construction of prognostic nomograms. In validation, we calculated the concordance index (C‐index) to evaluate the judgment ability of the nomograms. Receiver operating characteristics (ROC) curves and the area under the ROC curve (AUC) were applied to evaluate the sensitivity and specificity of nomograms. In general, C‐index and AUC value >0.7 indicate the satisfactory discriminative ability of the predictive tool.

All statistical analyses were performed with R (version 4.1.2) and SPSS (version 22). The R packages including “rms,” “foreign,” “survival,” “caret,” and “survivalROC” were used. *P* < 0.05 were considered statistically significant.

## 3 RESULTS

### 3.1 Characteristics of patients and disease

**FIGURE 1:**
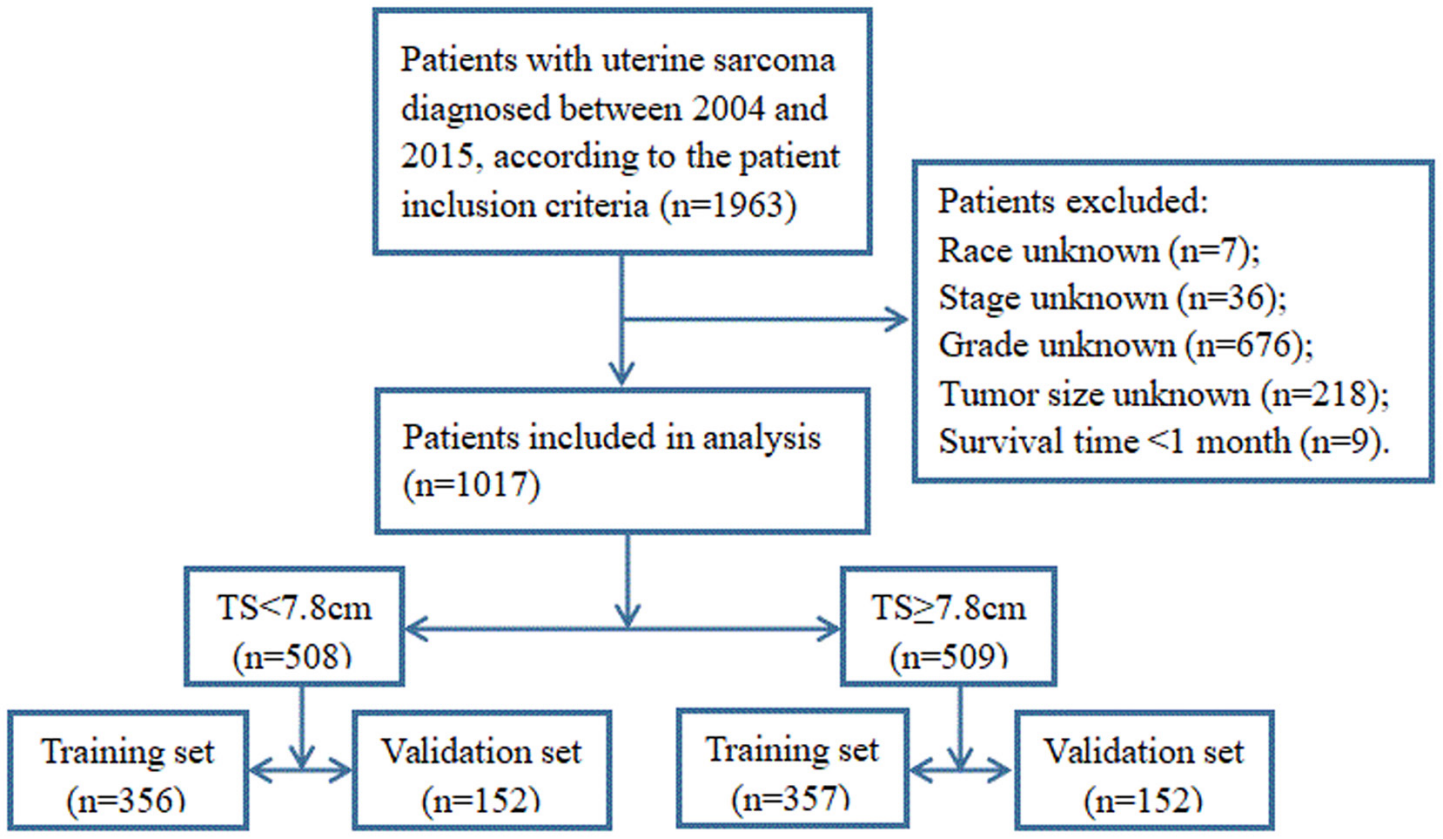
Flow diagram of patients selection from SEER database and constructing training and validation sets. SEER, Surveillance, Epidemiology, and End Results.

This study included 1017 patients diagnosed with uterine sarcoma from 2004 to 2015 in the SEER database (Figure 1). With regard to the features of patients in TS <7.8 cm group, nearly half of the patients were 45–64 years when diagnosed with uterine sarcoma. With regard to race, white women represented 71.26% of the patients. Regarding marital status, more than half of the patients were married. Regarding other pathological features, almost 3/4 of the patients had the tumor primary site in the endometrium, 98.03% had surgery on the primary site, 62.2% had localized tumor, and more than half had grade III‐IV histology. There were significant differences between TS <7.8 cm group and TS ≥7.8 cm group, including age, race, tumor primary site, stage, and grade. Compared with TS <7.8 cm group, TS ≥7.8 cm group had more patients of 45–64 years group (63.46% vs. 49.61%), higher black race prevalence (21.22% vs. 14.37%), higher proportion of myometrium tumor (37.72% vs. 13.97%), higher stage (regional and distant, 56.78% vs. 37.8%), and higher grade (III‐IV, 78.59% vs. 59.45%). All the patient demographics and clinicopathological features are presented in Table 1.

**TABLE 1:** The demographics and clinicopathologic characteristics of 508 patients with tumor size <7.8 cm and 509 patients with tumor size ≥7.8 cm

	TS <7.8 cm, *n* = 508		TS ≥7.8 cm, *n* = 509		x^2^	*P* value
	*n*	%	*n*	%		
Age					22.14	<0.0001*
<45 years	95	18.7	56	11		
45–64 years	252	49.61	323	63.46		
≥65 years	161	31.69	130	25.54		
Race					8.35	0.0154*
White	362	71.26	329	64.63		
Black	73	14.37	108	21.22		
Others^a^	73	14.37	72	14.15		
Marital status					7.47	0.1130
Married	265	52.17	253	49.71		
Single	92	18.11	121	23.77		
Divorced and Separated	70	13.78	73	14.34		
Widowed	55	10.82	46	9.04		
Unknown	26	5.12	16	3.14		
Primary site					88.07	<0.0001*
Endometrium	379	74.61	242	47.55		
Myometrium	71	13.97	192	37.72		
Fundus uteri	23	4.53	30	5.89		
Corpus uteri	35	6.89	45	8.84		
Surgery on primary site					0.67	0.4114
None	10	1.97	14	2.75		
Yes	498	98.03	495	97.25		
Stage					50.73	<0.0001*
Localized	316	62.2	220	43.22		
Regional	125	24.61	137	26.92		
Distant	67	13.19	152	29.86		
Grade					43.55	<0.0001*
Grade I–II	206	40.55	109	21.41		
Grade III–IV	302	59.45	400	78.59		

Abbreviation: TS, Tumor size.

^a^ Others included American Indian/AK Native, Asian/Pacific Islander.

* Statistically significant.

Both in TS <7.8 cm group and TS ≥7.8 cm group, approximately 70% of the patients were randomly assigned to a training cohort, respectively (TS <7.8 cm, *n* = 356; TS ≥7.8 cm, *n* = 357), and the rest patients of each group were randomly assigned to a validation cohort, respectively (TS <7.8 cm, *n* = 152; TS ≥7.8 cm, *n* = 152) (Figure 1). In each group, the training and validation cohorts were comparable in terms of patients' baseline characteristics (*P* > 0.05) (Table 2).

**TABLE 2:** Distribution of the assessed factors between the training and validation cohorts

	TS <7.8 cm				*P* value		TS ≥7.8 cm				*P* value
	Training cohort, *n* = 356		Validation cohort, *n* = 152				Training cohort, *n* = 357		Validation cohort, *n* = 152		
	*n*	%	*n*	%			*n*	%	*n*	%	
Age					0.0776						0.4126
<45 years	74	20.79	21	13.82			35	9.8	21	13.82	
45–64 years	178	50	74	48.68			229	64.15	94	61.84	
≥65 years	104	29.21	57	37.5			93	26.05	37	24.34	
Race					0.0783						0.2343
White	260	73.03	102	67.11			238	66.67	91	59.87	
Black	43	12.08	30	19.73			74	20.72	34	22.37	
Others^a^	53	14.89	20	13.16			45	12.61	27	17.76	
Marital status					0.5307						0.0790
Married	191	53.65	74	48.68			185	51.82	68	44.74	
Single	63	17.7	29	19.08			77	21.57	44	28.95	
Divorced and Separated	45	12.64	25	16.45			46	12.89	27	17.76	
Widowed	41	11.52	14	9.21			35	9.8	11	7.23	
Unknown	16	4.49	10	6.58			14	3.92	2	1.32	
Primary site					0.4289						0.9499
Endometrium	270	75.84	109	71.71			169	47.34	73	48.03	
Myometrium	44	12.36	27	17.76			137	38.38	55	36.18	
Fundus uteri	16	4.5	7	4.61			20	5.6	10	6.58	
Corpus uteri	26	7.3	9	5.92			31	8.68	14	9.21	
Surgery on primary site					0.7232						0.8500
None	6	1.69	4	2.63			9	2.52	5	3.29	
Yes	350	98.31	148	97.37			348	97.48	147	96.71	
Tumor size (cm)					0.9549						0.9843
TS <2	35	9.83	15	9.87		7.8 ≤ TS < 12	179	50.14	76	50	
2 ≤ TS <5	138	38.77	61	40.13		12 ≤ TS <22	156	43.7	66	43.42	
5 ≤ TS <7.8	183	51.4	76	50		TS≥22	22	6.16	10	6.58	
Stage					0.6892						0.7016
Localized	220	61.8	96	63.16			150	42.02	70	46.05	
Regional	91	25.56	34	22.37			98	27.45	39	25.66	
Distant	45	12.64	22	14.47			109	30.53	43	28.29	
Grade					0.6411						0.7321
Grade I–II	142	39.89	64	42.11			75	21.01	34	22.37	
Grade III–IV	214	60.11	88	57.89			282	78.99	118	77.63	

Abbreviation: TS, Tumor size.

^a^ Others included American Indian/AK Native, Asian/Pacific Islander.

### 3.2 Independent predictors for patients with uterine sarcoma

In the TS <7.8 cm training cohort, six variables (age, race, marital status, tumor primary site, stage, and grade) were identified as significantly associated with OS in multivariate analysis. ≥65 age (HR, 2.73; 95% CI, 1.33–5.63), black race (HR, 1.67; 95% CI, 1.03–2.72), single (HR, 1.69; 95% CI, 1.05–2.74), widowed (HR, 2.21; 95% CI, 1.35–3.60), corpus uteri tumor (HR, 1.88; 95% CI, 1.04–3.40), regional involved tumor (HR, 1.72; 95% CI, 1.15–2.58), distant involved tumor (HR, 3.6; 95% CI, 2.28–5.70), and high‐grade tumor (HR, 4.54; 95% CI, 2.61–7.89) were associated with worse OS (Table 3).

**TABLE 3:** Multivariate Cox regression analyses for overall survival in two training sets

Characteristics	TS <7.8 cm training set				TS ≥7.8 cm training set		
	HR	95% CI	*P* value		HR	95% CI	*P* value
Age							
<45 years	Ref.				Ref.		
45–64 years	1.83	0.94–3.55	0.0765		1.13	0.71–1.81	0.5982
≥65 years	2.73	1.33–5.63	0.0063*		1.52	0.91–2.55	0.1089
Race							
White	Ref.				Ref.		
Black	1.67	1.03–2.72	0.0395*		1.20	0.85–1.69	0.3046
Others^a^	1.25	0.78–2.00	0.3540		1.16	0.76–1.78	0.4835
Marital status							
Married	Ref.				Ref.		
Single	1.69	1.05–2.74	0.0319*		0.90	0.61–1.32	0.5815
Divorced and Separated	1.51	0.87–2.61	0.1418		1.14	0.76–1.70	0.5317
Widowed	2.21	1.35–3.60	0.0015*		1.50	0.97–2.33	0.0695
Unknown	0.84	0.30–2.39	0.7484		1.41	0.72–2.75	0.3113
Primary site							
Endometrium	Ref.				Ref.		
Myometrium	1.31	0.79–2.17	0.2927		1.08	0.81–1.44	0.6130
Fundus uteri	0.54	0.17–1.76	0.3073		1.27	0.69–2.33	0.4469
Corpus uteri	1.88	1.04–3.40	0.0359*		1.39	0.87–2.22	0.1688
Surgery on primary site							
None	Ref.				Ref.		
Yes	0.59	0.22–1.57	0.2919		0.22	0.11–0.46	<0.0001*
Tumor size (cm)							
TS <2	Ref.			7.8 ≤ TS < 12	Ref.		
2 ≤ TS <5	0.94	0.43–2.05	0.8800	12 ≤ TS < 22	1.27	0.96–1.68	0.0961
5 ≤ TS <7.8	1.32	0.63–2.80	0.4618	TS≥22	2.26	1.33–3.84	0.0027*
Stage							
Localized	Ref.				Ref.		
Regional	1.72	1.15–2.58	0.0083*		1.54	1.09–2.18	0.0146*
Distant	3.60	2.28–5.70	<0.0001*		3.41	2.45–4.75	<0.0001*
Grade							
Grade I–II	Ref.				Ref.		
Grade III–IV	4.54	2.61–7.89	<0.0001*		3.44	2.20–5.39	<0.0001*

Abbreviations: CI, confidence interval; HR, hazard ratio; Ref., Reference; TS, Tumor size.

^a^ Others included American Indian/AK Native, Asian/Pacific Islander.

* Statistically significant.

However in the TS ≥7.8 cm training cohort, only four variables (surgery on primary site, tumor size, stage, and grade) were significantly identified in multivariate Cox regression analysis. Surgery on primary site (HR, 0.22; 95% CI, 0.11–0.46) was an independent protective factor for OS. Tumor size ≥22 cm (HR, 2.26; 95% CI, 1.33–3.84), regional involved tumor (HR, 1.54; 95% CI, 1.09–2.18), distant involved tumor (HR, 3.41; 95% CI, 2.45–4.75), and high‐grade tumor (HR, 3.44; 95% CI, 2.20–5.39) were associated with worse OS (Table 3).

### 3.3 Construction and validation of nomograms

**FIGURE 2:**
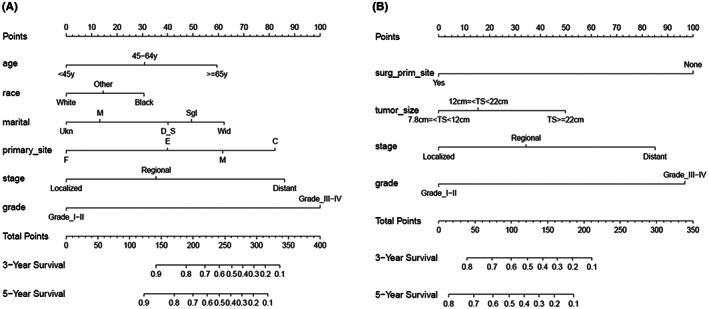
Nomograms predicting 3‐ and 5‐year overall survival of uterine sarcoma patients with tumor size <7.8 cm (A) and with tumor size ≥7.8 cm (B). Marital status: M, Married; Sgl, Single; D S, Divorced and Separated; Wid, Widowed; Ukn, Unknown; Primary site: E, Endometrium; M, Myometrium; F, Fundus uteri; C, Corpus uteri; TS, Tumor size; Surg prim site, Surgery on primary site; Other, included American Indian/AK Native, Asian/Pacific Islander.

Nomograms were constructed to predict survival both in the TS <7.8 cm training cohort and TS ≥7.8 cm training cohort. Independent prognostic factors in multivariate Cox models were applied to develop the nomograms for predicting 3‐ and 5‐year OS (Figure 2).

**FIGURE 3:**
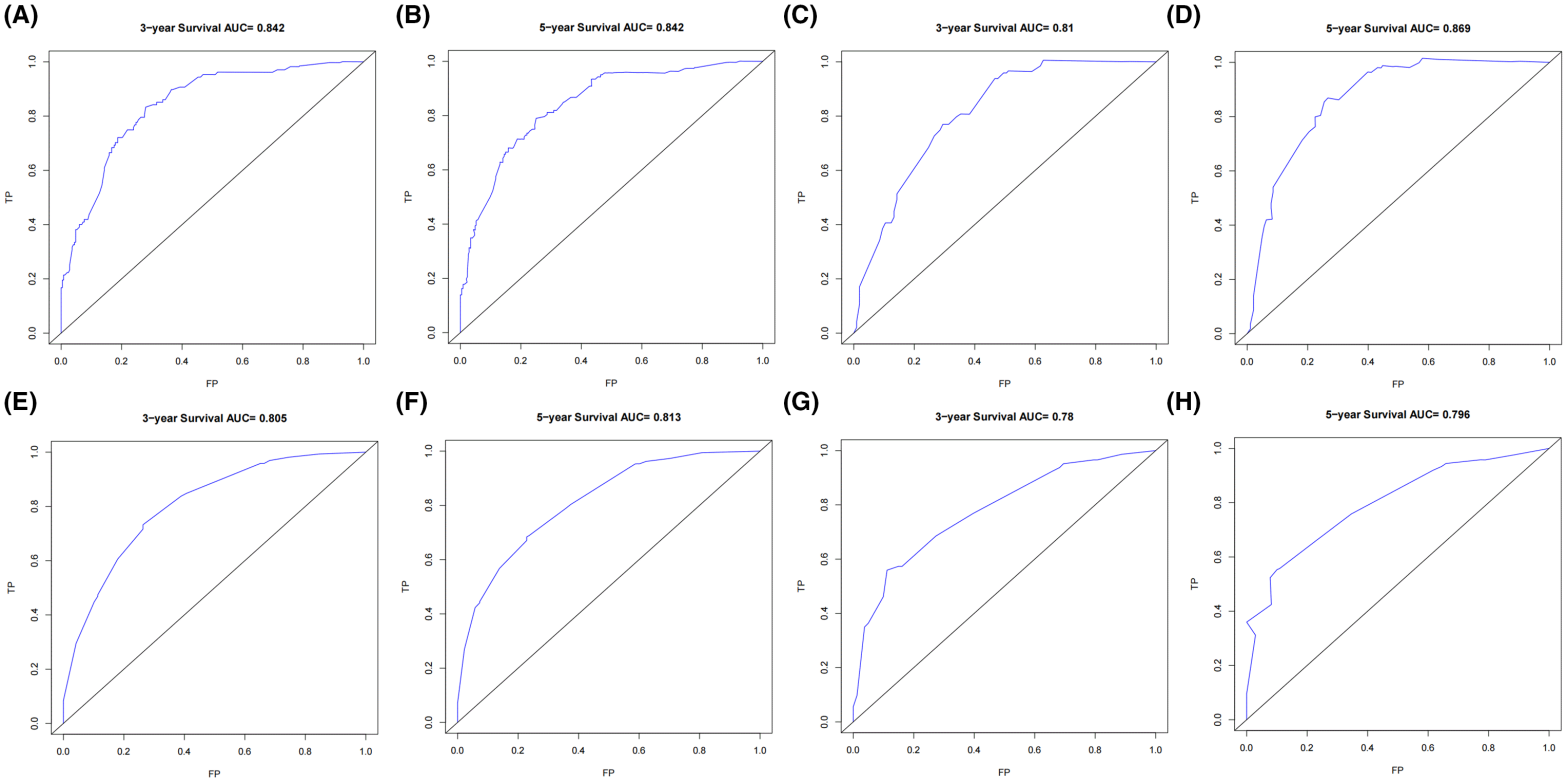
ROC curves and AUC values for 3‐ and 5‐year overall survival predictions in TS <7.8 cm training set (A, B) and validation set (C, D); ROC curves and AUC values for 3‐ and 5‐year overall survival predictions in TS ≥7.8 cm training set (E, F) and validation set (G, H). ROC, Receiver Operating Characteristic; AUC, Area Under Receiver Operating Characteristic Curve; TS, Tumor size; TP, True positive; FP, False positive.

With regard to the nomogram for patients with tumor size <7.8 cm, the C‐index value was 0.80 (95% CI, 0.77–0.83) in the training cohort and 0.78 (95% CI, 0.73–0.84) in the validation cohort. Regarding the nomogram for patients with tumor size ≥7.8 cm, the C‐index value was 0.73 (95% CI, 0.70–0.76) in the training cohort and 0.72 (95% CI, 0.66–0.77) in the validation cohort. In addition, for the nomogram in TS <7.8 cm group, the AUC values for 3‐ and 5‐year OS predictions in training and validation set were all above 0.80. For the nomogram in TS ≥7.8 cm group, the AUC values in training set were above 0.80, and in validation set were around 0.78 (Figure 3). These results suggested that the nomograms established in our study were useful for the prediction of survival in uterine sarcoma patients.

## 4 DISCUSSION

Patients with uterine sarcoma have poor prognosis, suggesting that comprehensive evaluation of their prognosis is critical. Thus far, the prognostic factors of uterine sarcoma are not fully identified, and further consensuses on the significance of some factors are needed. According to previous literature, current staging system for uterine sarcoma does not provide a sufficient prediction for clinical outcome. Uterine sarcomas are staged by using the FIGO 2009 staging system. In general, the stages of the FIGO staging system are closely related to OS of patients. However, some researchers observed different prognoses among patients at the same stage and speculated that this heterogeneity of prognosis may be because some important factors were not taken into account by the FIGO staging system.^6^ In recent years, nomograms have become more popular than traditional staging systems in predicting the prognosis of tumor patients. This is because the nomogram incorporates multiple characteristic variables, such as age, race, marital status, and tumor grade, into a quantitative model and can calculate the survival rate based on the individual characteristics of the patient.^15^ Nomogram has well promoted the development of personalized medicine. In a previous study, the researcher typically established one OS predicting nomogram for all tumor‐size groups of patients with uterine sarcoma. In our study, we found that the prognostic factors of survival were different between patients with larger tumors (≥7.8 cm) and those with smaller tumors (<7.8 cm). Therefore we established the tumor‐size‐stratified nomograms for uterine sarcoma patients, so as to improve the predictive performance. To the best of our knowledge, this research is the first to establish the tumor‐size‐stratified nomograms for uterine sarcoma patients. In our present study, the nomograms established for patients in two tumor size groups showed satisfactory discriminative ability. The nomograms demonstrated potential value in academic research and clinical practice.

Previous studies showed that the tumor size was correlated with OS rates in uterine sarcoma patients. However, there are few agreed‐upon cut‐offs for tumor size. For instance, Garg et al. demonstrated that the 5 cm was a better cut‐off.^16^ Another study showed that 8 cm or 10 cm tumor size was a significant prognostic indicator, but not 5 cm.^15^ In addition, the median tumor size of uterine sarcoma reported in the previous literature was mostly 7–9 cm,^17^, ^18^, ^19^ which was consistent with our finding (7.8 cm). Therefore, factoring in the appropriate cut‐off value and median tumor size, this study stratified the patients according to the tumor size of 7.8 cm. We found that TS ≥7.8 cm group had more patients in 45–64 years group, higher black race prevalence, higher proportion of myometrium tumor, higher stage, and higher grade. In particular, multivariate Cox regression analyses for OS showed that the effect of tumor size on patient's prognosis was different in different groups. In TS ≥7.8 cm group, we further divided the tumor size into 3 subgroups, and tumor size ≥22 cm was significantly associated with a worse prognosis. In general, the tumor size is positively correlated with the proliferation of the tumor. Larger tumors are more likely to invade surrounding blood vessels and lymph nodes, leading to tumor distant metastasis and poor prognosis. Meanwhile, it was shown that when patients with larger tumor size (≥7.8 cm), the surgery had the most powerful prognostic value for OS. On the contrary, surgery failed to show correlation with survival when patients with smaller tumor size (<7.8 cm). This should not be explained as meaning that surgery had no benefit on survival. In the present study, the majority of patients underwent surgery, making it difficult to properly evaluate the impact of surgery. Surgery is generally considered to be the best way to treat solid tumors. Some academics believe that surgery remains the standard treatment for uterine sarcoma.^20^ Furthermore, it is important to select a correct surgery approach. Meanwhile, tumor size failed to show an association with OS of patients with tumor size <7.8 cm. However, some demographic characteristics which were not independent predictors of OS in patients with larger tumor size showed their prognostic value of patients with tumor size <7.8 cm.

According to our findings, age ≥ 65 years, black race, single, widowed, and primary site in corpus uteri were significantly associated with poor prognosis when patients with smaller tumor size (<7.8 cm). A study on racial disparities in women with uterine cancer suggests that black race patients have more aggressive histological types and higher tumor grade when compared to white race patients.^21^ In addition, molecular differences and socioeconomic differences may also be important factors to interpret the racial disparity.^21^, ^22^, ^23^, ^24^ One of the possible reasons that single women have poor prognosis is that women's choice of treatment decisions is affected by marital status. For example, the results of one study showed that the unmarried group was less likely to receive radiotherapy, chemotherapy, and surgery than the married group.^25^ Moreover, researchers have shown that a supportive spouse may encourage his partner to develop healthy behaviors and lifestyles.^26^ In a retrospective study of patients with soft tissue sarcoma, Shilong Zhang et al. suggested that widowed patients had the highest death risks among the unmarried patients, and the unmarried patients tended to be diagnosed at an advanced stage.^27^ Several researches have analyzed the association between demographics and survival, but the results were inconsistent.^6^, ^28^ Due to the lack of research on the association between primary site of uterine sarcoma and survival, whether this factor has enough merit to be included in the overall nomogram schema requires more prospective data to support. Several studies suggested that tumor grade and stage were two strong predictors of survival,^8^, ^9^, ^29^, ^30^ which was consistent with our findings. In our study, both in larger (TS ≥7.8 cm) and smaller (TS <7.8 cm) tumor size groups patients with high tumor grade (grade III‐IV) or advanced tumor stage were considered to have worse prognosis.

Some other prognostic factors, although not included in this study, have been reported in other literature in recent years for their effects on disease prognosis. The selection of optimal surgical procedure and adjuvant therapy remains a dilemma in uterine sarcoma treatment.^28^ A retrospective study of 3650 patients indicated that the postoperative radiotherapy was able to improve local control of uterine sarcoma, but it did not have predictive value for OS.^31^ However, another study of 50 cases demonstrated postoperative radiation to be the significant prognostic factor for clinical outcome.^32^ A previous study reported that inflammatory markers were correlated with patients' prognosis. MJ Jeong et al. performed a multi‐institutional study and suggested that a raised preoperative neutrophil‐to‐lymphocyte ratio was associated with poor prognosis in patients with uterine sarcoma.^33^ Previous data indicated that high mitotic index was also associated with worse prognosis.^34^, ^35^ In addition, some biomarkers and hormonal treatment were also reported as potential prognosis factors in several researches.^36^, ^37^

Our study used SEER registry data, which are highly complete and accurate. Several limitations of this study should be acknowledged. Firstly, some other variables which may be effective supplements to the established nomograms need to be considered in future studies. Secondly, there were some selection bias, as the nomograms were established based on a retrospective study. Thirdly, the established nomograms required external validation to more comprehensively assess the applicability in the patients.

## 5 CONCLUSIONS

This study found that the significant prognostic factors were different between two tumor size groups of uterine sarcoma patients. For patients with relatively small tumor size, the independent prognostic factors of OS were age, race, marital status, tumor primary site, tumor stage, and grade. However, for patients with larger tumor size, the independent prognostic factors were surgery on primary site, tumor size, tumor stage, and grade. The tumor‐size‐stratified nomograms, which we constructed and validated, might be useful to predict the probability of survival for patients with uterine sarcoma. In addition, further refinements for these models are needed in future studies.

## AUTHOR CONTRIBUTIONS

Shiyu Cao and Shipeng Yan designed the study. Shiyu Cao, Shipeng Yan, Yingyun Hu, Xianzhen Liao, and Kekui Xu collected and analyzed the data. Haifan Xiao, Zhaohui Shi, Yanhua Zou, and Can Li checked the integrity of the data and the accuracy of the analysis results. Shiyu Cao wrote the manuscript. Shipeng Yan and Yingyun Hu revised the manuscript. All authors read and approved the final manuscript.

## ETHICS STATEMENT

No ethical approval was required or sought for the research because all the data (anonymized) analyzed in this research were collected from the SEER database with public available approach.

## Data Availability

Data availability statement The data in this research were retrieved from an open public database and can be accessed through this link: https://seer.cancer.gov/.
